# Correspondence

**Published:** 1885-10

**Authors:** Flavel S. Thomas

**Affiliations:** Hanson, Mass.


### CORRESPONDENCE.

*To the Editors of the* JOURNAL OF COMPARATIVE MEDICINE AND SURGERY:

GENTLEMEN—In the April number I started the degree ball, and yet it rolls. I hope it will not be allowed to stop until it is decided which is the best degree, and all schools have adopted it.

In the July number is an excellent article upon this subject, and I agreed almost entirely with the writer until I reached the last two lines, and I object to only four words in these two lines. He writes as follows: "There should be a convention of delegates from all these schools, and one title—Doctor of Veterinary Medicine—adopted by all."

If he had written "one title—Veterinary Surgeon—adopted by all," the ideas in the article would have been so nearly like my own that I could have imagined that I was the author of the article.

I object to D.V.S. or D.V.M. "B" writes: "The title one has is simply the announcement to the public that one is capable of offering it the services of an educated professional. The more we complicate this title by adding on a whole alphabet of letters, which are entirely meaningless to the public, the more we detract from the dignified position that we should assume."

Does not D.V.M. complicate this title? The public know us as Veterinary Surgeons; the public know what V.S. means.

How many people in America know what D.V.M. means? Not one in ten—perhaps, fifty—thousand. If we adopt D.V.M. we must teach the public that V.S. does not mean Veterinary Surgeon, and that D.V.M. does mean Veterinary Surgeon. This will take at least fifty years, and if even then the public see any reason for the change they will see more than I can now. "B" writes: "How many Americans know what F.R.C.V.S. means?" Many more than know what D.V.M. means. The writer wishes a short title with few letters; yet he throws aside V.S. for D.V.M.

Let us throw aside the "Doctor." There is no honor in it; every quack, vender of patent medicines, every blacksmith who has ever given a drench, every medium is called "Doctor." I have long since thought it a disgrace than an honor. The D.V.M is a "horse doctor," and he cannot deny it, but the V.S. can say "I am *not* a 'horse doctor;' I *am* a veterinary surgeon."

We read in the above-mentioned article as follows: "It would seem as if many of the younger men in the profession had gone mad in their desire to hide their regular calling under the more resonant title of 'Medical Doctor.'" . Is this not almost exactly what "B" advocates? Does he not ask that all schools confer the degree of (Veterinary) Medical Doctor?

I do not wish to give the impression that my object is to criticize this article, for I am much pleased with it. I only wish to convince the writer and others, that V.S. is a more appropriate title than D.V.M.

"B" writes, in speaking of veterinarians using M.D., "it required no such addition to his name to enable Bouley to become President of the Society of France. Gerlach, Leisering, Roell, Toussaient, Chauvaeux, and others needed no M.D. to lift them into the heaven of mortals."

Did they need a D.V.M.? Were they not simply Veterinary Surgeons?

Why is it not just as well for the schools to certify that one is a Veterinary Surgeon as to proclaim that one is a Doctor of Veterinary Medicine? V.S. is simple, well known, practical, terse; D.V.M. is ponderous, pretentious, unknown to the public, uselessly long (four words, instead of two), and it is meaningless to the public.

D.V.M. was first conferred by Cornell University, and only after six years of hard study, and now it is conferred by Harvard, and Iowa Agricultural College upon the completion of only three years of study.

One-half its value taken off at a stroke. It will soon be given upon the completion of two six months courses. In fact, this apparently is what "B" is working for, as he wishes all schools to adopt it.

But "B" and I are working [for the same thing; that is, one degree instead of half a dozen, and that one the most simple, practical and expressive.

Hanson, Mass. FLAVEL S. THOMAS, V.S.

